# Image-Based Finite Element Modeling Approach for Characterizing In Vivo Mechanical Properties of Human Arteries

**DOI:** 10.3390/jfb13030147

**Published:** 2022-09-11

**Authors:** Liang Wang, Akiko Maehara, Rui Lv, Xiaoya Guo, Jie Zheng, Kisten L. Billiar, Gary S. Mintz, Dalin Tang

**Affiliations:** 1 School of Biological Science and Medical Engineering, Southeast University, Nanjing 210096, China; 2 The Cardiovascular Research Foundation, Columbia University, New York, NY 10019, USA; 3 School of Science, Nanjing University of Posts and Telecommunications, Nanjing 210023, China; 4 Mallinckrodt Institute of Radiology, Washington University, St. Louis, MO 63110, USA; 5 Department of Biomedical Engineering, Worcester Polytechnic Institute, Worcester, MA 01609, USA; 6 Mathematical Sciences Department, Worcester Polytechnic Institute, Worcester, MA 01609, USA

**Keywords:** finite element updating approach, arterial material properties, in vivo, material parameters estimation

## Abstract

Mechanical properties of the arterial walls could provide meaningful information for the diagnosis, management and treatment of cardiovascular diseases. Classically, various experimental approaches were conducted on dissected arterial tissues to obtain their stress–stretch relationship, which has limited value clinically. Therefore, there is a pressing need to obtain biomechanical behaviors of these vascular tissues in vivo for personalized treatment. This paper reviews the methods to quantify arterial mechanical properties in vivo. Among these methods, we emphasize a novel approach using image-based finite element models to iteratively determine the material properties of the arterial tissues. This approach has been successfully applied to arterial walls in various vascular beds. The mechanical properties obtained from the in vivo approach were compared to those from ex vivo experimental studies to investigate whether any discrepancy in material properties exists for both approaches. Arterial tissue stiffness values from in vivo studies generally were in the same magnitude as those from ex vivo studies, but with lower average values. Some methodological issues, including solution uniqueness and robustness; method validation; and model assumptions and limitations were discussed. Clinical applications of this approach were also addressed to highlight their potential in translation from research tools to cardiovascular disease management.

## 1. Introduction

Mechanical forces play a fundamental role in the initiation, development and final critical clinical events of cardiovascular diseases (CVD) such as stroke and heart attack [1,2]. As diseases progress, biochemical compositions of cardiovascular tissues alter as well as their mechanical properties [3,4]. Clinical observations have shown that elevated tissue stiffness associated with pathology often represents an early warning sign of diseases, as in atherosclerosis [4], heart failure [5] and even cancer diseases [3]. Therefore, accurate determination of the mechanical properties of the arterial wall could provide meaningful information for cardiovascular research in multifaceted ways: (a) estimating the material stiffness of the vascular tissues to assess the severity of cardiovascular diseases such as atherosclerosis [6]; (b) being an essential element of the computational models to simulate biomechanical conditions for better understanding cardiovascular physiology and pathophysiology such as stress-based aortic aneurysm rupture risk assessment [7]; (c) searching for plausible substitutes with proper mechanical properties to replace diseased arterial segments, such as tissue engineering vascular grafts [8]; (d) predicting the mechanical interactions between arteries and implanted devices for better treatment prognosis [9]; and many other applications. 

Great effort has been exerted to perform ex vivo experiments on human arterial walls in health and disease, and important conclusions on their mechanical behaviors were drawn [10,11,12]. Ex vivo experiments includes inflation-extension testing, indentation testing, uniaxial extension and planar biaxial testing [13,14,15]. These experiments record the deformation responses (stretch, strain, displacement or elongation) of the arterial tissues corresponding to given loading conditions (stress, force or pressure). Several constitutive models were proposed to fit these experimental stress–strain or stress–stretch ratio data [11]. These theoretical and experimental analyses have deepened our understanding on the elastic mechanical properties of vascular tissues. Early experimental studies on healthy arteries showed that the stress–stretch ratio curve of the cardiovascular tissue was typically in exponential form. Therefore, a Fung-type-material model was proposed to describe the material properties for these tissues [10]. To study the mechanical properties of diseased tissues, Holzapfel et al., examined atherosclerotic plaque tissues in the iliac artery ex vivo using uniaxial testing [16]. Experimental data indicated that tissue properties were highly nonlinear and anisotropic. An anisotropic Mooney–Rivlin material model was introduced to describe the mechanical properties of atherosclerotic plaques [11]. Furthermore, layer-specific and component-specific material properties of carotid plaque were also documented, and a large inter-specimen variation was reported [17]. Location-specific material properties along the aortic segments were also systematically investigated, and Peña et al., reported that the healthy aortic tissue became more anisotropic and stiffer as the distance to the heart increased [18]. The experimental data collected from these ex vivo studies are fundamental to formulating and testing the constitutive models. Excellent reviews on these experimental methods could be found in the literature [13,14,15]. 

Even though considerable ex vivo experimental data have accumulated over the years, they only provided biomechanical information of arterial diseases at one time-point, which is very limited for clinical applications for two reasons: (a) ex vivo arterial tissues isolated from the vascular tree might not represent the exact mechanical properties as in the living subjects [19]; (b) characterization of patient-specific tissue material properties in vivo is more suitable for monitoring arterial stiffness over the long term for disease management, and predicting mechanical responses of the arteries when medical devices are implanted [6]. Therefore, there is a pressing need to characterize patient-specific tissue material properties in vivo. 

This review paper aims to review the studies to characterize subject-specific arterial wall properties in vivo using a finite-element-model-based updating approach (FEMBUA). Material properties of the aorta, carotid and coronary arteries determined from the novel FEMBUA and classical ex vivo experimental methods were compared to investigate whether any discrepancy in material properties exists for both approaches. Section 2 will present other simple methods to characterize arterial tissue material properties in vivo other than the FEMBUA. The comprehensive framework of the FEMBUA and its elaborate procedure will be detailed in Section 3. Section 4 will report the mechanical properties of different arterial walls, including aorta, carotid and coronary, published in in vivo studies, and their results will be further compared to those from ex vivo experimental studies. Section 5 will discuss some methodological issues with respect to the FEMBUA, followed by the conclusion remarks and future directions in Section 6. 

## 2. In Vivo Methods to Quantify Material Properties of Arterial Walls 

Several in vivo methods were proposed to characterize subject-specific material properties of arterial walls in vivo or even in situ. The principle of these methods is to acquire the arterial wall deformation and corresponding loading conditions from clinical data, and link them to obtain the arterial material properties. Thanks to the considerable advances in medical imaging technologies, dynamic vessel motion can be recorded in vivo via time-resolved imaging modalities, such as ultrasound (US), cine magnetic resonance imaging (MRI), cine intravascular ultrasound (IVUS) and electrocardiogram (ECG)-gated computed tomography (CT). Blood pressure conditions are also measured as the loading conditions to drive the vessel to pulsating deformations. Simple and sophisticated methods were proposed in the existing literature based on different assumptions. 

Early in vivo studies of vessel material properties assumed that the arterial wall was a straight thin-walled circular cylinder with linear elastic material properties, and its stiffness could be estimated by Peterson’s modulus (denoted as Ep) using the formula [19,20]:

Ep = ∆P × d/∆d(1)

where d is the diameter, and ∆P and ∆d are the differences in pressure and diameter at diastole and systole, respectively. This method is very intuitive and provides a simple way to measure arterial stiffness using pressure and strain data. However, a more rigorous index for material stiffness is called Young’s modulus (E), which is the ratio of stress over strain. Formula (1), together with Laplace’s law, is employed to calculate E as [21]:

E = ∆P × d2/2h∆d ( = d/2h × Ep)(2)

where h is the wall thickness. 

Another simple and commonly used formula to estimate the Young’s modulus of the arterial wall is the Moens–Korteweg equation, which relates the Young’s modulus of the blood vessel with pulse wave velocity (PWV) from the heart to the peripheral vascular [20]:

(3) $$\mathrm{PWV}=\sqrt{\mathrm{Eh}/\rho d}$$

where ρ is the density of the blood. More details on the derivation of the abovementioned formulas can be found in [20]. These simple in vivo methods have already been applied into clinical settings to assess the risk of cardiovascular diseases [22,23]. However, they rely on strong assumptions without considering arterial thickness and geometry, nonlinear anisotropic elastic properties, tissue heterogeneity and location-specific material property variations. 

To obtain more sophisticated nonlinear anisotropic mechanical behavior of arterial walls, an analytical approach was employed with constitutive models incorporated to characterize their nonlinear stress–strain relationship by identifying the material parameters in the constitutive models [24]. In this approach, material parameters of the constitutive models could be determined as follows: (1) pressure and vessel deformation were measured simultaneously from the individuals in clinical practice; (2) classical solid mechanics theory was used to establish the relationship between vessel stress and pressure analytically to obtain stress conditions using measured pressure conditions [24,25,26]; (3) parameter values in selected constitutive models would be chosen to fit vessel stress and deformation data. However, this approach treated the blood vessel as ideal circular geometry, and was based on classical solid mechanics theory (like Laplace’s law) which excluded the discrepancy of deformation in the arterial tissue. This simplification would lead to inaccurate calculations of pressure estimation and stress/strain distributions in the arteries, and wipe out the local stress concentration, which are closely related to atherosclerotic plaque rupture and aneurysm rupture. To overcome this limitation, a finite-element-model-based updating approach (FEMBUA) was introduced by several groups to quantify the complex mechanical properties of patient-specific arterial tissues. The following section will introduce the sophisticated framework of this approach. 

## 3. Framework of Finite-Element-Model-Based Updating Approach

**Figure 1:**
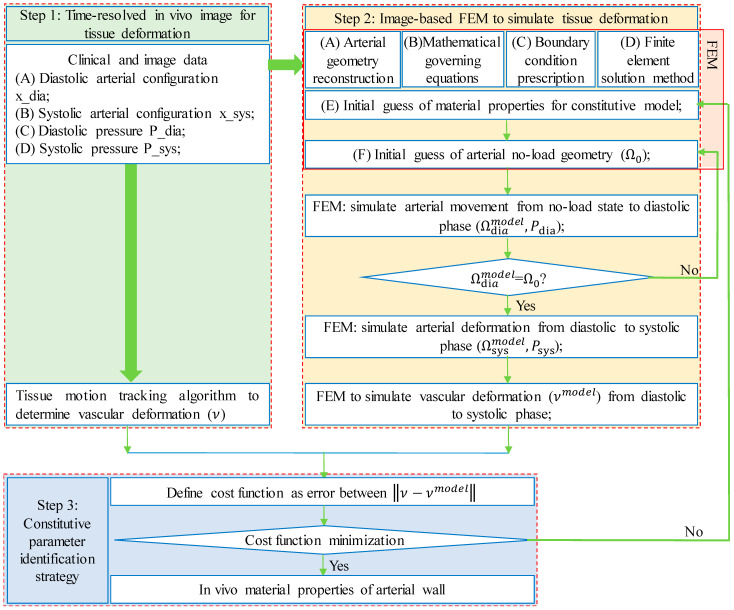
Flowchart of finite-element-model-based updating method to identify the in vivo material properties based on clinical data at diastolic and systolic phases using deformation as criterion.

The in vivo quantification of mechanical properties of arterial tissues based on in vivo medical images is intrinsically an inverse problem [27,28]. The steps of the FEMBUA to solve the inverse problem are outlined below (Figure 1): 

Step 1, time-resolved in vivo medical image acquisition with tissue deformation under dynamic loading conditions; 

Step 2, image-based FEM to simulate the tissue deformation corresponding to in vivo loading conditions with tissue material properties to be determined; 

Step 3, constitutive parameter identification strategy to find the correct tissue material properties so that tissue deformation in FEM would recover that on in vivo images. Full descriptions of these steps are given in the following subsections. 

### 3.1. Data Acquisition and Vessel/Tissue Motion Tracking

In Vivo Clinical Data Acquisition: To visualize the vascular deformation in vivo, time-resolved imaging modality was employed to obtain a series of medical images to track arterial deformation during one cardiac cycle. Table 1 summarizes some commonly used imaging technologies to detect vessel motion for various arterial walls in the clinical setting. More specifically, time-resolved 3D ultrasound (t+3D US) [29] and ECG-gated CT [30] were used to track aortic tissue motion with acceptable image resolution. Cine-MRI [31] and Cine-IVUS [32] were used to obtain carotid and coronary motion data, respectively. Besides the image data, simultaneous loading conditions, such as on-site pulsating blood pressure waveform, were also acquired. Noninvasively measured arm cuff blood pressures were obtained in some studies in lieu of on-site blood pressure to avoid invasive procedures [32]. Other loading information such as external compression force and active stress from smooth muscle cells cannot be estimated in vivo. These forces were normally not considered in FEM [26]. 

Vessel/Tissue Motion Tracking: Arteries deform under time-varying loading conditions. One simple way to approximate the deformation is to calculate the changes in lumen circumference, lumen area or lumen volume between two cardiac phases and consider it as vessel deformation under two different pressure conditions. Typically, diastolic and systolic phases were selected to quantify the deformation of the arterial wall, with the diastolic phase often treated as the “reference” phase and systolic phase as the deformed phase (see Figure 1). This way, we could quantify the average deformation of the arterial wall by treating it as a homogeneous material. However, more sophisticated methods, such as the speckle tracking algorithm [39] and digit image correlation [40], were introduced to track regional tissue displacement by examining the cross-correlation of the speckle patterns in the medical images from diastolic to systolic phases. The accuracy of these algorithms has been validated in vivo and in vitro, with good agreements found [29]. More details on the algorithms could be found in these excellent reviews [40,41]. Franquet et al., also investigated vessel material properties with more cardiac phases taken into consideration [37]. 

### 3.2. Image-Based Finite Element Models

In the FEMBUA, image-based finite element models (FEMs) are constructed to calculate plaque stress/strain conditions, while the parameter values in those models are determined iteratively so that model solutions can satisfy measured vessel/tissue deformation conditions. The FEM models constructed to identify the material properties of the arterial wall in the literature were mainly structure-only models to expedite the updating approach detailed in Section 3.3. The essential elements to construct such a model contain the following procedures: 

Arterial Wall Geometry Reconstruction: 3D US, CT, MRI and IVUS images can show the cross-section of the arterial wall, and the images corresponding to the diastolic phase were used to reconstruct the referenced geometry of the arterial wall [34,42,43]. For some imaging modalities, some heterogeneous components (e.g., atherosclerotic plaque compositions, intraluminal thrombus) can also be detected and reconstructed for more accurate representation of vessel wall structure [43]. 

Pre-Stressing Geometry Estimation: Since medical images were acquired in vivo, the referenced geometry reconstructed were loaded with the physiological pressure on the luminal surface, axially stretched, and other loading conditions (such as circumferential residual stress). Thus, a pre-stressing algorithm should be performed to obtain no-load geometry (corresponding to zero-pressure condition) based on the referenced geometry as the initial geometry to start the computational simulation. To this end, Guo et al., employed a pre-shrink stretch procedure by shrinking the referenced coronary artery geometry circumferentially and axially to obtain the no-load geometry [32]. Speelman et al., proposed a backward incremental method to estimate the arterial geometry under the no-load state [44]. Other patient-specific algorithms were also developed and can be found in the relevant reference [45]. It should be noted that the no-load geometry should be estimated with the prerequisite of known material properties of the arterial wall. However, since the arterial material properties are unknown at this stage, no-load geometry would be determined along with in vivo arterial material properties following the constitutive parameter identification strategy specified in Section 3.3. 

Constitutive Models for Arterial Wall: Arterial walls are generally treated as elastic, either anisotropic or isotropic, nearly-incompressible, homogeneous material. They could also be considered as heterogeneous material if different tissue components were included [46]. Several constitutive models were proposed to describe their mechanical properties ranging from the simple Hookean model to the more sophisticated nonlinear anisotropic ones [11,47]. A list of commonly used material models including the Hookean model [31], NeoHookean model [48], Yeoh model [49], Demiray model [30], Mooney–Rivlin (MR) model [11], Gasser–Ogden–Holzapfel (GOH) model [50], Holzapfel2005 model [51], Fung-type material [10,52] and their strain energy density functions are provided in the supplement (see Supplementary File). The constitutive parameters in the material models were to be determined following the strategy in Section 3.3. 

Mathematical Equations Governing Arterial Wall Motion: The mathematical equations governing arterial wall motion consist of equations of motion, strain-displacement relations and the stress–strain relations that could be derived from strain energy density functions for hyperelastic materials [53]. With a proper prescription of boundary conditions, this equation system could be solved to obtain vessel biomechanical conditions, such as arterial wall deformation and stress/strain conditions, which would be used to compare with corresponding clinical measurements. 

Boundary Conditions: Proper boundary conditions were applied to FEM to mimic the loading conditions on the arterial wall in vivo. The most important loading condition that triggers vascular deformation is pulsating pressure prescribed on the luminal surface on the arterial wall [42]. It corresponds to the differential blood pressure measured from each individual between the referenced state and the deformed state. In addition to the pressure conditions, some other loading conditions, such as external pressure conditions and axial stretch, were also considered in some studies for more accurate simulations [24,26]. Proper fixity boundary conditions should be applied to avoid unexpected rigid body movement of the arterial wall [54]. 

Solution Method for the Finite Element Model: Finite element mesh could be generated using commercial finite element software such as ANSYS, ADINA, Abaqus or self-developed in-house software. FEM models were further solved using sophisticated numerical schemes built in these software. Mesh analysis should be performed to guarantee the accuracy of the model solution, which would affect the accuracy of material parameter estimation [42]. 

### 3.3. Constitutive Parameter Identification Strategy

To obtain vessel material properties correctly, vessel deformation from FEM should match those from in vivo medical images [55]. A cost function was introduced to measure the discrepancy between arterial wall deformation from FEM and from in vivo images. Then, an optimization algorithm was utilized to find the optimal material parameters along with the no-load vessel geometry by minimizing the cost function. 

Cost Function Definition: Most studies constructed the cost function as the sum of the squares of the nodal deformation differences between FEM vessel morphology and vessel geometry reconstructed from images [34,36]. Some studies also simply defined the cost function as the square difference in lumen circumference or lumen area or lumen volume [32]. These measurements are more available, and easier to calculate. However, the drawback is that these measurements only provide one quantity from one FEM, so arterial tissues must be assumed to be uniform homogeneous materials. Variations of the cost functions also exist by comparing the difference in pressure or stress from FEM and from clinical measurements in prior studies [26,36]. 

Optimization Algorithm to Search Correct Material Properties: The value of the cost function abovementioned is dependent on the material parameters in FEM models. It is a nonlinear, multivariate optimization problem to find the correct constitutive parameters by minimizing the cost function [28,29], especially in the case considering the arterial wall as heterogeneous material [56]. The number of material parameters increases linearly as the arterial wall is divided into several subdomains with different material parameters. 

There are two essential difficulties [24] inherent in this type of nonlinear and nonconvex optimization problem: (1) the cost function has multiple local minima, and simple gradient-based algorithms may not be able to find a global minimum; (2) a second more fundamental difficulty is over-parameterization, and solutions of material parameters may not be unique. To address these difficulties, a combined stochastic/deterministic approach was recommended in some studies with a two-step approach: Step 1, hundreds of sets of material parameters were chosen randomly by the Monte Carlo algorithm to evaluate the cost function; Step 2, a deterministic nonlinear algorithm, such as the Nelder–Mead simplex algorithm [57], was applied to obtain the final material parameters, using the parameter set with the minimal cost function value determined from Step 1 as initial parameters. 

Once the constitutive parameters were found, a stress–strain (or stress–stretch ratio) relationship could be derived from the strain energy density functions. More details on the derivation can be found in the existing literature and are omitted here [11,18,52]. For comparison purpose, the effective Young’s modulus was defined as the slope of the proportional function to fit nonlinear stress–stretch ratio material curves on the stretch interval [1.0 1.1] to measure the tissue stiffness [58]. For anisotropic material models, effective Young’s moduli were calculated for the material curves along both the circumferential and longitudinal directions (by fixing the stretch ratio to 1.0 in the other direction) and denoted as Ec and Ea, respectively. 

## 4. In Vivo Mechanical Properties of Individual-Specific Arterial Wall Tissue

The framework of the FEMBUA was employed to determine individual-specific material properties of arterial tissues for mostly middle- or large-size arteries in vivo [59]. To examine the difference in material properties obtained from the FEMBUA and classical experimental approaches, some representative ex vivo experimental studies were selected, and their results were compared with those from in vivo studies using the FEMBUA. Table 2 lists some prior studies on human aorta, carotid and coronary arteries using this in vivo identification approach, as well as some ex vivo experimental studies for comparison purpose. Subject information of these in vivo and ex vivo studies is also provided. Furthermore, one representative set of material parameters (average values or median values of the material parameters for all samples from each study) were chosen to plot the material curves and calculate the effective Young’s modulus. More details on each vascular bed are given in the following subsections. 

### 4.1. Aortic Tissue

**Figure 2:**
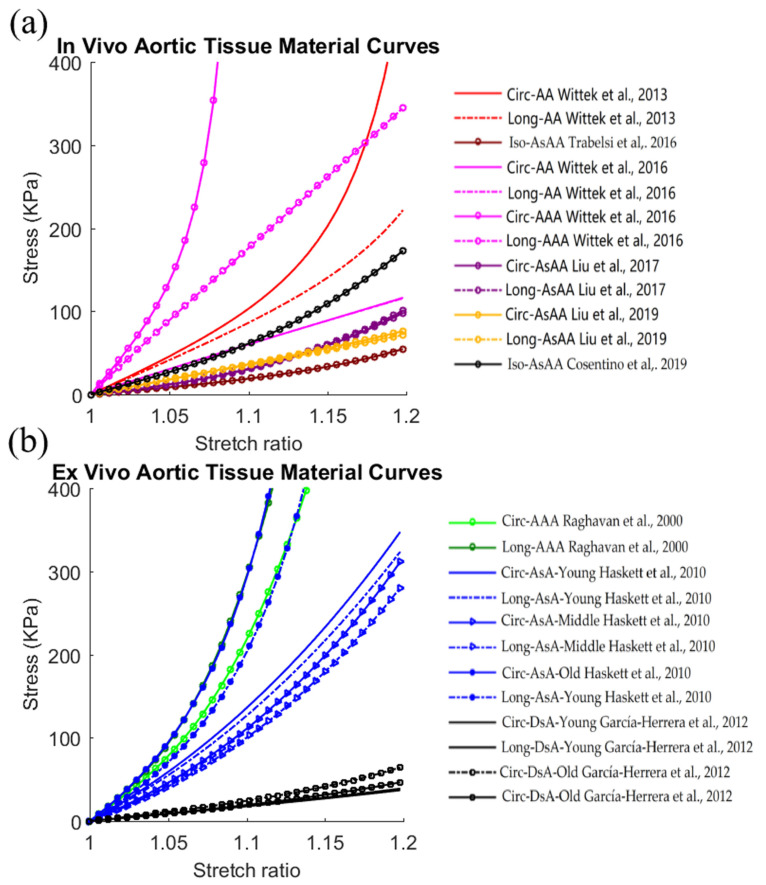
Stress–stretch ratio curves of healthy and diseased aortic tissues from (**a**) in vivo studies and (**b**) ex vivo studies. Abbreviations: AA, abdominal aorta; AAA, abdominal aortic aneurysm; AsA, ascending thoracic aorta; AsAA, ascending thoracic aortic aneurysm; DsA: descending thoracic aorta; Ec, effective Young’s modulus in circumferential direction; Ea, effective Young’s modulus in longitudinal direction; Iso, isotropic material; Circ, material curves in circumferential direction; Long, material curves in longitudinal direction [29,30,33,34,35,49,55,60,61].

The human aorta contains a wide range of vascular course, including ascending thoracic aorta (AsA), aortic arch, descending thoracic aorta (DsA) and abdominal aorta (AA). Aortic aneurysm is a common pathological condition influencing the health state of the aorta. The stress–stretch ratio curves of healthy and aneurysmal aortic tissues listed in Table 2 using the in vivo FEMBUA method and ex vivo experimental methods are plotted in Figure 2. Material curves in the circumferential and longitudinal directions were given for anisotropic material models. 

Based on the material curves in Figure 2a,b, large variations in aortic tissue stiffness could be observed for both in vivo studies and ex vivo studies. For in vivo studies, the Ec ranged from 180.3 kPa to 5576.7 kPa (Ea from 180.3kPa to 1770.2 kPa) whereas in ex vivo studies, the Ec ranged from 181.5 kPa to 2382.4 kPa (Ea from 176.0 kPa to 1856.3 kPa). This demonstrated that the aortic tissue stiffness values from in vivo and ex vivo studies were in the comparable ranges. However, compared to in vivo studies, all aortic tissues (including healthy and diseased tissues) from ex vivo studies yielded higher average tissue stiffness weighted by number of samples, with 201.5% higher stiffness for the Ec (2040.4 kPa vs. 676.9 kPa), and 267.1% higher for the Ea (1922.8 kPa vs. 523.8 kPa), respectively). It could also be observed that most listed studies had higher tissue stiffness in the circumferential direction than in the longitudinal direction [49]. Moreover, tissue anisotropy was clear but less significant in ex vivo studies, according to the difference in circumferential and longitudinal material curves from the same study [29,34,60]. 

Among the listed in vivo studies, one study determined the material properties of both healthy and aneurysmal tissues to study the impact of pathological conditions on the material properties following the same method. This study showed that aneurysmal aortic tissues tend to have higher stiffness than non-aneurysmal aortic tissues [33]. This conclusion was consistent with other ex vivo studies [66]. Furthermore, it is fortunate that García-Herrera et al. [61] and Haskett et al. [60] harvested enough specimens from healthy donors to investigate the aging effect on tissue material properties. García-Herrera et al. [61] reported that aortic stiffness increased as the age increased. For the study from Haskett et al. [60], they classified the donors into young, middle age and old groups. They found that the oldest people had the highest aortic stiffness, whereas middle-age donors had close but slightly lower aortic stiffness than the young group. 

### 4.2. Carotid and Coronary Arterial Tissues

**Figure 3:**
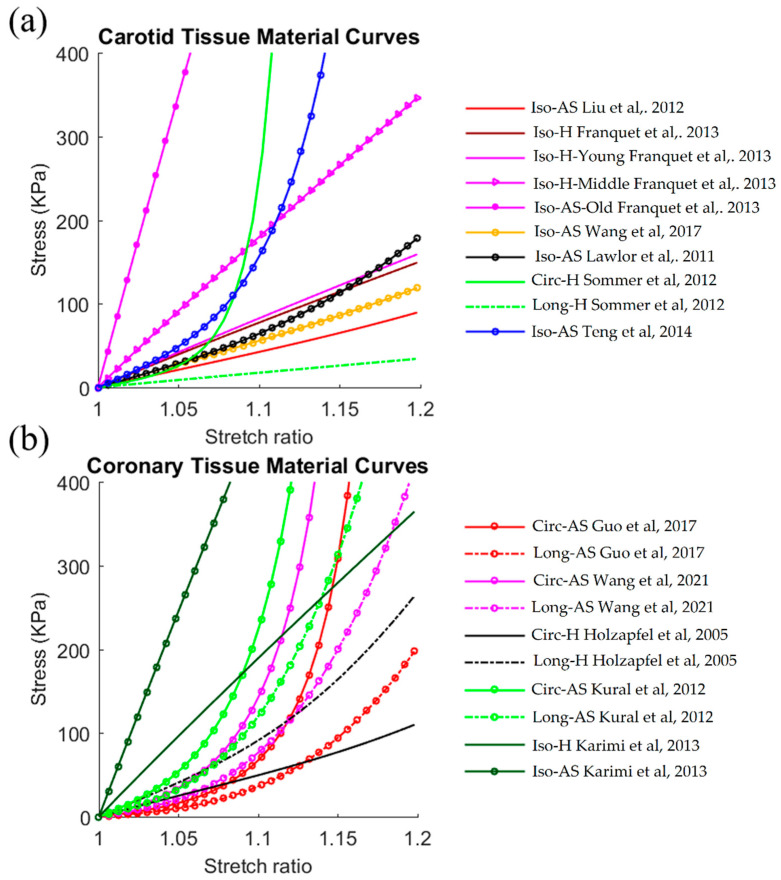
Stress–stretch ratio curves of healthy and diseased aortic tissues for all (**a**) carotid studies and (**b**) coronary studies. Abbreviations: Iso, isotropic material; Circ, material curves in circumferential direction; Long, material curves in longitudinal direction; H, healthy; AS, atherosclerosis [17,31,32,36,37,38,51,58,62,63,64,65].

Fewer studies were performed to identify the material properties of carotid and coronary arteries in vivo. Figure 3 gives the plots of the material curves of healthy and diseased carotid and coronary tissues listed in Table 2. These studies have shown similar results to aortic tissue. Compared to aortic studies, tissue anisotropy was more obvious in carotid and coronary specimens, with the Ec higher than the Ea for the listed studies. All carotid studies in Table 2 showed that the Ec and Ea both ranged from 422.6 kPa to 6926.2 kPa for in vivo studies whereas the Ec ranged from 606.2 kPa to 1245.4 kPa (Ea from 176.7 kPa to 1245.4 kPa) for ex vivo studies. Therefore, tissue stiffness values from in vivo studies were generally in the same magnitude as that from ex vivo studies. Coronary studies yielded a similar conclusion. Similarly, compared to ex vivo studies, carotid/coronary tissues from ex vivo studies yielded a higher average tissue stiffness weighted by number of samples. For carotid tissues, the average tissue stiffness values from ex vivo and in vivo studies were 1137.6 kPa vs. 849.2 kPa (34% higher) for the Ec, and 999.0 kPa vs. 849.2 kPa (17.6% higher) for the Ea, respectively. For coronary tissues, the numbers were 1975.2 kPa vs. 973.6 kPa (102.9% higher) for the Ec, and 1959.6 kPa vs. 562.3 kPa (248.5% higher) for the Ea, respectively. Atherosclerosis is the most common disease occurring in both carotid and coronary arteries. This disease elevates the tissue stiffness in carotid and coronary, as demonstrated by Franquet et al. [37] and Kaimi et al. [65]. This phenomenon was also observed by ex vivo studies [52]. An aging effect was also investigated by Franquet et al. [37], and their results demonstrated an increase in the elastic modulus of the common carotid artery as age increases.

## 5. Some Methodological Issues in Finite-Element-Model-Based Updating Approach

### 5.1. Significance of In Vivo Identification Framework

Classically, mechanical experiments are conducted to quantify the mechanical properties using arterial tissues ex vivo [14]. However, the FEMBUA provides another way to determine tissue properties in vivo. This in vivo method could be easily modified to successfully apply to other biological tissues, and even non-biomaterials such as cerebral aneurysmal tissue [67]; cardiac tissue [68]; thigh muscle tissue [69]; human skin [70]; silicone gel soft tissue [71]; and metal [72]. 

Since tissue samples are often not available for in vivo studies and the material properties of arterial tissues alter when taken out of living subjects, the image-based finite element modeling approach is more suitable for studies under in vivo conditions with potential clinical implementations [22,23]. Prior studies have demonstrated that patient-specific in vivo tissue material properties had a significant influence on cardiovascular biomechanics, especially in strain calculation compared to ex vivo material properties [38]. Therefore, patient-specific in vivo material properties are desirable for personalized treatment. 

Even though in vivo and ex vivo methods follow different approaches in determining tissue properties, they are not exclusive to each other. Rather, they are complementary to each other. A hybrid approach that uses a finite element updating approach to match stress–strain data from biaxial/uniaxial experiments has been proposed to quantify the aortic aneurysmal tissue [73]. 

### 5.2. Comparison in Tissue Stiffness from FEMBUA and Ex Vivo Experimental Approaches

To assess its accuracy and efficacy, comparison analysis between the novel FEMBUA and classical ex vivo experimental approaches were performed. Some representative ex vivo studies were selected, and the tissue stiffness values from these studies were compared to those from in vivo studies using the FEMBUA. For all types of arteries (aorta, carotid or coronary), the ranges of tissue stiffness were generally in the same magnitude for both methods. However, the dissected tissues from ex vivo studies were stiffer than tissue samples from in vivo studies by average. It should be noted that the limited number of ex vivo studies could influence the conclusions abovementioned, because the average tissue stiffness would change if a different set of ex vivo representative studies were chosen. Thus, the comparison was evaluated numerically, not statistically for all tissues, not for just healthy or diseased ones, in which case, the selected studies would have more influence. Nevertheless, these conclusions suggested that the FEMBUA is an accurate and effective approach for quantifying the material properties of arterial walls. However, more rigorous comparison analysis should be performed by conducting both methods on the same tissue samples as detailed in the following section. 

### 5.3. Validation of In Vivo Identification Approach

Several research groups investigated vessel material properties using both the in vivo FEMBUA method and ex vivo experiments on the same arterial tissue for validation purposes [30,35,55]. Based on five aortic aneurysmal specimens from two patients, Liu M et al., compared the mechanical properties of aortic tissue from in vivo and ex vivo biaxial testing methods. The authors found that material curves from both methods were close to the average value of the mean absolute percentage error less than 5% [35]. Based on a larger sample size (n = 10), Cosentino et al., reported similar observations. They stated that at strain of 0.14, the relative difference in stress response from the stress–strain curves of both methods was less than 24% [55]. Additionally, Trabelsi et al., performed FEMs with the material parameters determined from both in vivo and ex vivo methods for the same tissue. Their simulation showed that the difference in peak wall stress between the two methods was less than 20% [30]. These studies supported that, using ex vivo experiment testing as the gold standard, the FEMBUA yields mild difference in biomechanical results for the same specimen. 

### 5.4. Method Reproducibility and Noise Sensitivity Analysis

Reproducible, accurate determination of arterial tissue material properties is an important prerequisite for clinical applications. In a methodological study, Narayanan et al., demonstrated the reproducibility of the FEMBUA by repeating the approach for three patients to obtain their material properties. The results of material parameters from different runs were recovered with errors of 3.0 ± 4.7% [28]. 

The robustness of the solution to the inverse problem remains to be an important issue. There are many uncertainties that would impact the results of mechanical property determination which include medical image resolution, on-site pressure measurement, etc. Narayanan et al., performed sensitivity analysis by applying random Gaussian noise to the original medical images. Their results showed that the errors for the material parameters were 1.3 ± 1.6% for the 5% noise addition. In addition to the noise inherent to the image-based geometry, Narayanan et al., also investigated the impact of such pressure perturbations on material property recovery. They claimed that such perturbation would result in controllable error in tissue stiffness estimation. More specifically, the relative error was equal to the relative error of the applied perturbation [28]. 

### 5.5. Modeling Assumptions and Limitations

There are some assumptions involved in FEM for in vivo indentation of the material properties of arterial walls, which would impact the results from the FEMBUA: (a) Axial stretch has considerable impact on the result of mechanical parameter values [38,48]. Therefore, patient- and vessel-specific axial stretch data are needed to determine more accurate material properties. Currently, due to a lack of in vivo axial stretch data, prior studies just set it to a given stretch ratio in the computational models when determining in vivo material properties. Wang et al. [38] investigated the impact of axial stretch on material properties. Their results indicated that smaller axial stretch led to greater slice shrinkage and softer material stiffness estimation. (b) Neglecting perivascular pressure conditions could lead to over- or under-estimation of material properties, depending on vascular bed. That is because it is the transmural pressure, not blood pressure alone, that drives the arteries to expand and contract. Due to the perivascular tissue and environment that the aorta, carotid and coronary arteries are situated in, perivascular pressure conditions are different, and physiologically not equal to zero [26]. Not considering a positive perivascular pressure in the FEM would lead to an overestimation of the material stiffness, whereas ignoring a negative one would lead to underestimation. (c) Active stress from smooth muscle cells has an impact. The function of the smooth muscle in the arterial wall is to produce active tension, relatively independent of stretch. However, its impact on arterial stiffness remains controversial. Early evidence supported that the contribution of smooth muscle cell to the elastic properties of living blood vessel was very small [74]. More recently, Tremblay et al., considered the active stress in their method to estimate the material properties, and claimed that the effect of smooth muscle cell activation was non-negligible and could increase both the circumferential and axial stiffness of the tissue [75]. More attempts have to be made to set down the role of active stress played in tissue mechanical properties. (d) Residual stress was not included as no patient-specific opening angle data were available [76,77]. Currently, there are no studies that includes residual stress in their computational finite element models to characterize arterial wall properties. More efforts are needed to understand the impact of residual stress on results of mechanical properties. (e) Structure-only models instead of fluid-structure interaction models were typically utilized in the FEMBUA, because it is more computationally efficient, especially when multiple iterations were needed to obtain the optimal material properties in the updating approach. Furthermore, prior studies have demonstrated that structure-only models could close biomechanical conditions to the fluid–structure interaction models [53]. 

## 6. Conclusions Remarks and Future Directions

The image-based FEMBUA has been proven to accurately and effectively determine the material properties of arterial walls in vivo. In addition, the tissue stiffness from this method was consistent with that from ex vivo experimental approaches. However, current studies mainly use image and pressure data at two cardiac phases (typically systolic and diastolic phases). Imaging technologies with higher temporal resolution are desirable to obtain clinical images at more cardiac phases, so that more data points will be available to fit the complex nonlinear material models (such as the anisotropic Mooney–Rivlin model or Fung-type model with several material constants) in a least square sense. Thus, the image-based FEMBUA would be more robust and not sensitive to image noise or pressure measurement error. 

Biomechanical properties provide vast information regarding cardiovascular tissues in living individuals, which could guide us to a better understanding of arterial mechanics and physiology, as well as for the analysis of the mechanisms of vascular diseases. 

In vivo arterial tissue stiffness has already been employed in clinical settings as a risk factor for cardiovascular diseases, and has been proven to have clinical significance [22,23]. Since the FEMBUA could provide more detailed information on mechanical properties under in vivo conditions, it holds great potential in clinical applications for personalized treatment and precision medicine: (1) sophisticated material properties from this method, rather than simple arterial stiffness, are essential to accurately estimate stress/strain conditions for possible clinical applications, such as a stress-based diagnosis strategy to refine current diameter-based diagnosis criteria in aortic aneurysm assessment [7,78]; and (2) in vivo identification of the nonlinear anisotropic material properties of the cardiovascular tissue is also a prerequisite for predicting its interaction with implanted devices, such as coronary stents. Now, patient-specific computational modeling of coronary stents has been performed for individualized pre-procedural planning and predicting stenting prognosis [79]. Large-scale clinical studies are needed to verify the efficiency of in vivo material properties for clinical decision-making in these applications. Lastly, successful applications of computational modeling incorporating in vivo mechanical properties are based on the solid ground of an automated implementation of this in vivo approach with computational efficiency. With further validations, the FEMBUA could be further developed and automated to provide vessel material properties, which are an essential part for arterial models.

## Figures and Tables

**Table 1:** Selected time-resolved image modalities to visualize human vascular motion and deformation in clinical setting. Abbreviations: Time-resolved 3D ultrasound (t+3D US); electrocardiogram (ECG)-gated computed tomography (ECG-gated CT); cine magnetic resonance imaging (MRI); cine intravascular ultrasound (IVUS).

Image Modality	Temporal Resolution	Spatial Resolution	Artery	Strength and Weakness in Arterial Wall Detection	Reference
t + 3D (4D) US	~10 frames/s	~0.5 mm	Aorta	Cheap, fast and easy way to detect arterial boundaries and tissue compositions, but inter- and intra-observer variability in image interpretation;	[29,33]
ECG-gated CT	~10 frames/cardiac cycle	~0.5 mm	Aorta	Superb calcified tissue detection and lumen detection; limited in detecting other plaque compositions, such as lipid and vessel wall;	[30,34,35]
Cine MRI	~50 frames/cardiac cycle	~0.6 mm	Carotid	Detection of the whole vascular cross-section with superior soft-tissue contrast, but long scanning time;	[31,36,37]
Cine IVUS	~30 frames/s	100 µm	Coronary	High resolution and large penetration depth for arterial tissue detection, also can detect arterial tissue compositions;	[32,38]

**Table 2:** Subject information, study details and mechanical properties results from in vivo and some representative ex vivo studies. Abbreviations: AA, abdominal aorta; AAA, abdominal aortic aneurysm; AsA, ascending thoracic aorta; AsAA, ascending thoracic aortic aneurysm; DsA: descending thoracic aorta; Ec, effective Young’s modulus in circumferential direction; Ea, effective Young’s modulus in longitudinal direction.

Reference	Tissue Sample Information	Material Model	Imaging/Experiment Techniques	Effective Young’s Modulus
In Vivo Aorta				
[29]	5 AA samples from 5 healthy subjects	GOH model	t + 3D US	Ec = 969.5 kPa Ea = 843.7 kPa
[30]	5 AsAA samples from 5 patients	Demiray model	ECG-gated CT	Ec = Ea = 180.3 kPa
[33]	1 AA sample from 1 healthy subject	GOH model	t + 3D US	Ec = 605.7 kPa Ea = 605.4 kPa
	1 AAA sample from 1 patient			Ec = 5576.7 kPa Ea = 1770.2 kPa
[34]	4 AsAA samples from 4 patients	GOH model	ECG-gated CT	Ec = 270.2 kPa Ea = 276.5 kPa
[35]	4 AsAA samples from 4 patients	GOH model	ECG-gated CT	Ec = 363.1 kPa Ea = 355.7 kPa
[55]	9 AsAA samples from 9 patients	Yeoh model	ECG-gated CT	Ec = Ea = 573.9 kPa
Ex Vivo Aorta				
[49]	69 AAA specimens	Yeoh model	Uniaxial testing	Ec = 2382.4 kPa Ea = 1856.3 kPa
[60]	6 AsA specimens from donors with age 0 to 30	GOH model	Biaxial testing	Ec = 1268.4 kPa Ea = 1182.1 kPa
	6 AsA specimens from donors with age 31 to 60			Ec = 1025.5 kPa Ea = 905.9 kPa
	17 AsA specimens from donors with age above 61			Ec = 2365.8 kPa Ea = 1698.6 kPa
[61]	5 DsA specimens from 5 young donors with age 20 to 36	MR model	Uniaxial testing	Ec = 181.5 kPa Ea = 176.0 kPa
	5 DsA specimens from 5 old donors with age 45 to 60			Ec = 232.0 kPa Ea = 186.5 kPa
In Vivo Carotid				
[36]	12 atherosclerotic carotid samples from 12 patients	MR model	Cine MRI	Ec = Ea = 422.6 kPa
[31]	2 carotid samples from 2 healthy subjects	Hookean model	Cine MRI	Ec = Ea = 781.8 kPa
[37]	4 carotid samples from 4 young healthy subjects with age 24 to 26	Hookean model	Cine MRI	Ec = Ea = 833.7 kPa
	5 carotid samples from 5 middle-age healthy subjects with age 51 to 63			Ec = Ea = 1815.3 kPa
	4 atherosclerotic carotid samples from 4 old patients with age 68 to 76			Ec = Ea = 6926.2 kPa
[58]	81 atherosclerotic carotid samples from 8 patients	MR model	Cine MRI	Ec = Ea = 555.1 kPa
Ex Vivo Carotid				
[62]	14 atherosclerotic carotid specimens from 14 patients	Yeoh model	Uniaxial testing	Ec = Ea = 606.2 kPa
[63]	11 common carotid specimens from 11 relatively healthy subjects	Hozapfel2005 model	Extension-inflation tests	Ec = 1235.7 kPa Ea = 176.7 kPa
[17]	59 atherosclerotic carotid specimens of fibrous cap	MR model	Uniaxial testing	Ec = Ea = 1245.4 kPa
In Vivo Coronary				
[32]	2 atherosclerotic coronary samples from 1 patient	MR model	Cine IVUS	Ec = 484.6 kPa Ea = 279.8 kPa
[38]	20 atherosclerotic coronary samples from 13 patients	MR model	Cine IVUS	Ec = 1022.5 kPa Ea = 590.6 kPa
Ex Vivo Coronary				
[51]	13 coronary intima specimens from 13 relatively healthy subjects	Hozapfel2005 model	Uniaxial testing	Ec = 497.5 kPa Ea = 862.6 kPa
[64]	4 coronary specimens from 2 relatively healthy subjects	MR model	Biaxial testing	Ec = 1602.5 kPa Ea = 925.3 kPa
[65]	14 healthy coronary specimens	Hookean model	Uniaxial testing	Ec = Ea = 1909.5 kPa
	8 atherosclerotic coronary specimens			Ec = Ea = 4864.1 kPa

## Data Availability

Not applicable.

## Supplementary Materials

The following supporting information can be downloaded at: https://www.mdpi.com/article/10.3390/jfb13030147/s1.
