# Supplementary material for: Image-Based Finite Element Modeling Approach for Characterizing In Vivo Mechanical Properties of Human Arteries
Source: J Funct Biomater. 2022 Sep 11;13(3):147. doi: 10.3390/jfb13030147 (PMC9505727; doi:10.3390/jfb13030147)
Supplement: Supplementary file 1 [file jfb-13-00147-s001.zip › jfb-1906801-supplementary.pdf]

Image-Based Finite Element Modeling Approach for Characterizing In Vivo Mechanical Properties of Human Arteries

Liang Wang[1], Akiko Maehara[2], Rui Lv[1], Xiaoya Guo[3], Jie Zheng[4], Kisten L. Billiar[5], Gary S. Mintz[2], Dalin Tang[1,6,*]

Supplementary File:

Different tissues could exhibit various mechanical properties, linear vs nonlinear, isotropic vs anisotropic, and etc. Several material models were proposed in the existing literature to capitulate the stress-strain relationship of the arterial wall tissues, most of which are formulated as the strain energy density function. Some of the material models are phenomenological, like Fung-type model, isotropic Mooney-Rivlin material model, and others are based on arterial microstructure, like anisotropic Mooney-Rivlin material model. The mathematical equations for these are listed below:

A. Hookean material model:

The stress-strain relationship of Hookean or linear elastic material simply follows the Hooke's law:

$$\boldsymbol{\sigma} = \boldsymbol{C}\boldsymbol{\varepsilon}$$

where $\boldsymbol{\sigma}$ and $\boldsymbol{\varepsilon}$ are Lagrangian stress and strain tensors, while $\boldsymbol{C}$ is a fourth-order tensor, usually called the stiffness tensor or elasticity tensor (Fung, 1993).

B. NeoHookean material model:

The strain energy density function (denoted as W) of NeoHookean material model could be written as:

$$\mathrm{W} = \mathrm{c}_1(I_1 - 3)$$

where I_1 is the first invariants of right Cauchy-Green deformation tensor C defined as $C=[C_{ij}]=X^TX$, $X=[X_{ij}]=[\partial x_i/\partial a_j]$, (x_i) is deformed position, (a_i) is reference position.

C. Yeoh material model:

The strain energy density function of Yeoh material model is a polynomial form of I_1 with degree of 3, whose formula is:

$$W = c_1(I_1 - 3) + c_2(I_1 - 3)^2 + c_3(I_1 - 3)^3$$

D. Demiray material model:

The strain energy density function of Demiray material model is an exponential form of I_1:

$$W = D_1[\exp\big(D_2(I_1 - 3)\big) - 1]$$

E. Isotropic Mooney-Rivlin material model:

The strain energy density function of this model is:

$$W = c_1(I_1 - 3) + c_2(I_2 - 3) + D_1[\exp\big(D_2(I_1 - 3)\big) - 1]$$

where I_2 is the second invariants of right Cauchy-Green deformation tensor C. This model contains linear function and exponential function of I_1. Therefore, NeoHookean material model and Demiray material model could be considered as special cases for Isotropic Mooney-Rivlin material model.

E. Anisotropic Mooney-Rivlin material model:

The strain energy density function of anisotropic Mooney-Rivlin material model contains two terms: isotropic term and anisotropic term. The formula of isotropic term is the same as isotropic Mooney-Rivlin material model while the anisotropic term is accounting for the stretching of collagenous fibers.

$$W = W_{iso} + W_{aniso}$$

$$W_{iso} = c_1(I_1 - 3) + c_2(I_2 - 3) + D_1[\exp\big(D_2(I_1 - 3)\big) - 1]$$

$$W_{aniso} = \frac{K_1}{K_2}[\exp(K_2(I_4 - 3)^2) - 1]$$

where $I_4 = C_{ij}(n_c)_i(n_c)_j$, n_c is the unit vector in the circumferential direction of the vessel. Normally there are two set of fibers in arterial wall. Here we assume the two set of collagenous fibers have angles of $\pm\ \theta$ with the circumferential direction of arterial wall.

F. Gasser-Ogden-Holzapfel (GOH) model:

Gasser et al. employed one parameter κ to account for the tissue anisotropy, and proposed this material model with following strain energy density function (Gasser et al., 2006):

$$W = \frac{c_1}{2}(I_1 - 3) + \frac{K_1}{K_2}[\exp(K_2(\kappa I_1 + (1 - 3\kappa)I_4 - 1)^2) - 1]$$

G. Holzapfel2005 model:

The strain energy density function of the material model introduced by Holzapfel in 2005 is (Holzapfel et al., 2005):

$$W = \mu(I_1 - 3) + \frac{K_1}{K_2}[\exp(K_2[(1 - \rho)(I_1 - 3)^2 + \rho(I_4 - 1)^2]) - 1]$$

H. Fung-type model:

The strain energy density function of the Fung-type material model is the exponential form of the quadratic function of the terms in Green strain tensor E defined as $E = \frac{1}{2}(C - I)$:

$$W = \frac{c}{2}(\exp(Q) - 1)$$

Where $Q(E) = a_1 E_{11}^2 + a_2 E_{22}^2 + 2a_3 E_{11} E_{22}$.
